# Recovery of Seed Quality and Germination Success Following Severe Wildfires in *Araucaria araucana* Forests of the Southern Andes

**DOI:** 10.1002/ece3.74015

**Published:** 2026-07-23

**Authors:** Andrés Fuentes‐Ramírez, Bernardita Díaz‐Mons, Rodrigo Vargas‐Gaete, Héctor Herrera, Leonardo Almonacid

**Affiliations:** ^1^ Laboratorio de Ecosistemas y Bosques (EcoBos), Facultad de Ciencias Agropecuarias y Medioambiente Universidad de La Frontera Temuco Chile; ^2^ Centro Nacional de Excelencia para la Industria de la Madera (CENAMAD) Pontificia Universidad Católica de Chile Santiago Chile; ^3^ Center for Biodiversity and Ecological Sustainability (C‐BEST), Facultad de Ciencias Agropecuarias y Medioambiente Universidad de La Frontera Temuco Chile; ^4^ Programa de Magíster en Manejo de Recursos Naturales, Facultad de Ciencias Agropecuarias y Medioambiente Universidad de La Frontera Temuco Chile

**Keywords:** forest recovery, germination curves, monkey puzzle tree, post‐fire reproduction, seed viability, survival, wildfires

## Abstract

Increasing frequency and severity of forest fires is affecting natural ecosystems on a global scale. Temperate forests that have experienced severe wildfires are likely to encounter difficulties to recover and establish new cohorts of plants in the short‐term after fire. For *Araucaria araucana* old‐growth forests in the southern Andes, quantitative germination rates have been rarely reported after wildfires, yet this information is crucial for understanding the post‐fire reproductive potential in this iconic species of high ecological and cultural significance for global south ecosystems. We evaluated the germination of the long‐lived conifer *A. araucana* (monkey puzzle tree) 8 years after severe fires in Andean forests of south‐central Chile, using seeds collected within a National Reserve fire‐affected in 2015. We collected seeds from focal female trees initially surveyed in 2016 and evaluated their germination under controlled greenhouse conditions using Kaplan–Meier estimators. We then compared these results with germination rates obtained 1 year after the wildfire in the same area. Results revealed that germination significantly improved over time, rising from 65% 1 year after the fire to 90% 8 years post‐fire. This higher germination was also associated with increased seed viability of seeds 8 years post‐fire, as well as faster germination rates over time. We address the potential effect of heat in the early development of seeds collected 1 year after the fire, which may explain their lower viability and germination capacity, compared with seeds collected 8 years post‐fire. In summary, these findings demonstrate that, in absence of recurrent disturbances, surviving *A. araucana* trees exhibit a notable capacity for maternal recovery, highlighting its relevance for forest recovery in the southern Andes and the critical need to protect post‐fire landscapes from additional human and livestock pressures to allow these internal biological processes to secure the forest's reproductive future and long‐term resilience.

## Graphical abstract

We assessed post‐fire germination of *Araucaria araucana* in the southern Andes and found that seed viability and germination increased markedly (from 65% to 90%) between 1 and 8 years after a severe wildfire. Improved germination at longer time‐since‐fire reflects a recovery of reproductive capacity in this ecologically and culturally significant species. These results underscore the importance of protecting burned forests during early post‐fire years to support natural regeneration.

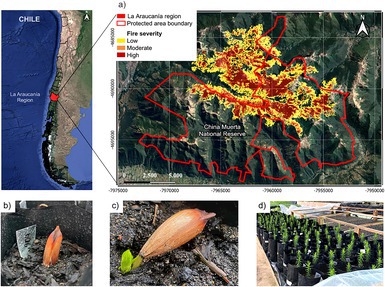

## 1 Introduction

Global evidence shows that fire can profoundly alter forest regeneration dynamics, with germination responses varying widely across species, fire regimes, and environmental conditions (Arroyo‐Vargas et al. 2022; Leal‐Medina et al. 2026). Many serotinous pine species and other conifers exhibit enhanced germination following fire due to heat‐triggered cone opening, reduced competition, and positive nutrient pulses (Keeley and Zedler 1998; Schwilk and Ackerly 2001; Pausas and Keeley 2017). In contrast, species lacking serotiny or thick protective bark often experience reduced post‐fire seed availability or low germination success, particularly after high‐severity fires that damage seed banks or maternal trees (Agee 1996). Climate anomalies have amplified these patterns by increasing fire severity and frequency, altering seed viability, and reshaping post‐fire microclimates essential for seedling establishment (Stevens‐Rumann et al. 2018; Davis et al. 2019). These altered physical environments exacerbate the vulnerability of recovering stands, where interactions with drought and herbivory can further limit plant regeneration, contributing to shifts from forests to alternative vegetation states in several regions worldwide (Rother and Veblen 2016; Coop et al. 2020). These global patterns highlight the complex nonlinear responses of conifers to fire, underscoring the importance of examining how key species, such as long‐lived *Araucaria araucana* (Mol.) K. Koch [monkey puzzle tree], respond to changing disturbance regimes (Arroyo‐Vargas et al. 2019; Fuentes‐Ramírez et al. 2019).

Natural temperate forests dominated by *A. araucana* and *Nothofagus* spp. in Chile and Argentina (hereafter termed as *Araucaria‐Nothofagus* forests) contain species with markedly different levels of fire tolerance (Cóbar‐Carranza et al. 2014; Almonacid‐Muñoz et al. 2024). Some species are highly susceptible to fire due to their thin bark and lack of resprouting capacity (e.g., *Nothofagus dombeyi* and *N. pumilio* ), while others exhibit fire‐adaptive traits such as thick bark, shedding of lower branches and the ability to resprout after fire (e.g., *A. araucana* ; Burns 1993; González et al. 2010; Díaz‐Mons et al. 2025). This pattern suggests that fire in these forests historically has been infrequent and generally of low‐to‐moderate severity (González et al. 2010; Fuentes‐Ramírez et al. 2022). However, recent increases in both fire frequency and severity in recent decades indicate a clear shift in the fire regime of these ecosystems (Fuentes‐Ramírez et al. 2020; McWethy et al. 2018).

The slow growing southern South American endemic conifer *A. araucana* is distributed between 37° and 40° S, mostly in the Andean region of south‐central Chile and Argentina (Veblen 1982; Sanguinetti et al. 2023). The species is currently protected under CITES, and it is listed as endangered by the IUCN (Premoli et al. 2013). It can reach heights of up to 50 m, with a diameter exceeding 2 m and a lifespan of over 1000 years (Aguilera‐Betti et al. 2017). Moreover, the species holds significant ecological and sociocultural importance for the local Pehuenche communities (Rozzi et al. 2012). However, it remains unclear whether the physiological stress from severe wildfires impairs the reproductive quality of surviving trees, or if *A. araucana* exhibits a recovery trajectory in seed viability and germination success as time since fire increases. To our knowledge only one study has documented an immediate negative effect of fire on the germination of *A. araucana* in the Andean range (Fuentes‐Ramírez et al. 2019), where seed viability and germination are lower than average, historical reproductive capacity (Sanguinetti 2014). It is also unknown whether maternal vigor and germination rates of this species can improve with time since fire, a factor that would be critical for prompting the regeneration and ensuring post‐fire recovery. To date, quantitative germination rates after fire have been rarely reported for *A. araucana* in Chile, where the most pristine and well‐conserved forests of the species are found.

*Araucaria araucana* exhibits substantial interannual variability in seed production (e.g., masting), as cone initiation and development are strongly influenced by climatic conditions such as temperature anomalies, precipitation regimes, and extreme weather events (Sanguinetti and Kitzberger 2009; Burns 1993; González et al. 2010). Warmer or drier conditions during key reproductive phases can markedly reduce seed availability (Veblen 1982; Sanguinetti et al. 2023; Aguilera‐Betti et al. 2017), which is critical for successful regeneration in fire‐affected landscapes (Peters et al. 2005). Following severe wildfires, suitable microsites for recruitment may be limited by altered soil properties, including increased exposure, reduced moisture availability, and high seed predation pressure (Fuentes‐Ramírez et al. 2019; Díaz‐Mons et al. 2025). Under such conditions, years with naturally low seed output can further constrain post‐fire seedling establishment, compounding the negative effects of disturbance (Stewart et al. 2021; Barbizan‐Sühs et al. 2021; Hacket‐Pain et al. 2024). Conversely, years with higher seed production may enhance the chances of recovery, but only when climatic conditions and the altered post fire environmental filters allow seeds to germinate, persist, and survive the early stage of establishment (Holz and Veblen 2011; Kitzberger et al. 2016). Given ongoing climatic shifts in south‐central Chile and Argentina, the interactions between climate driven reproductive variability and post‐fire conditions provide a critical lens for understanding the future regenerative capacity of *A. araucana* under increasingly frequent and severe disturbance regimes (McWethy et al. 2018; Fuentes‐Ramírez et al. 2020).

Having updated information regarding *A. araucana* germination is essential for understanding the potential reproduction of this iconic, ecologically and culturally significant species after fire (dos Reis et al. 2014), which can provide valuable insights into the establishment of seedlings and new cohorts of plants in severely burned forests both in the short‐ and mid‐to‐long‐terms. Thus, this study was specifically designed to address the following questions: (1) Do seed viability and the germination rate of *A. araucana* exhibit an improvement with increasing the time since fire in burned forests? and (2) How does this compare with the germination observed immediately after fire? We hypothesized that the initial reduction in seed performance observed immediately after fire would be reversed as maternal vigor recovers, leading to higher germination rates 8 years post‐fire disturbance. Hence, this study aims to quantify the recovery of *A. araucana* reproductive potential by comparing seed viability and germination performance at two distinct intervals following severe wildfire. By evaluating these parameters 8 years post‐fire and comparing them to baseline data from 2016, we seek to determine if maternal recovery significantly influences seed quality over time. Establishing these empirical baselines is a necessary step to provide the quantitative evidence required for assessing post‐fire forest dynamics and informing restoration strategies in fire‐affected Andean landscapes.

## 2 Material and Methods

### 2.1 Study Area and Wildfire Description

**FIGURE 1:**
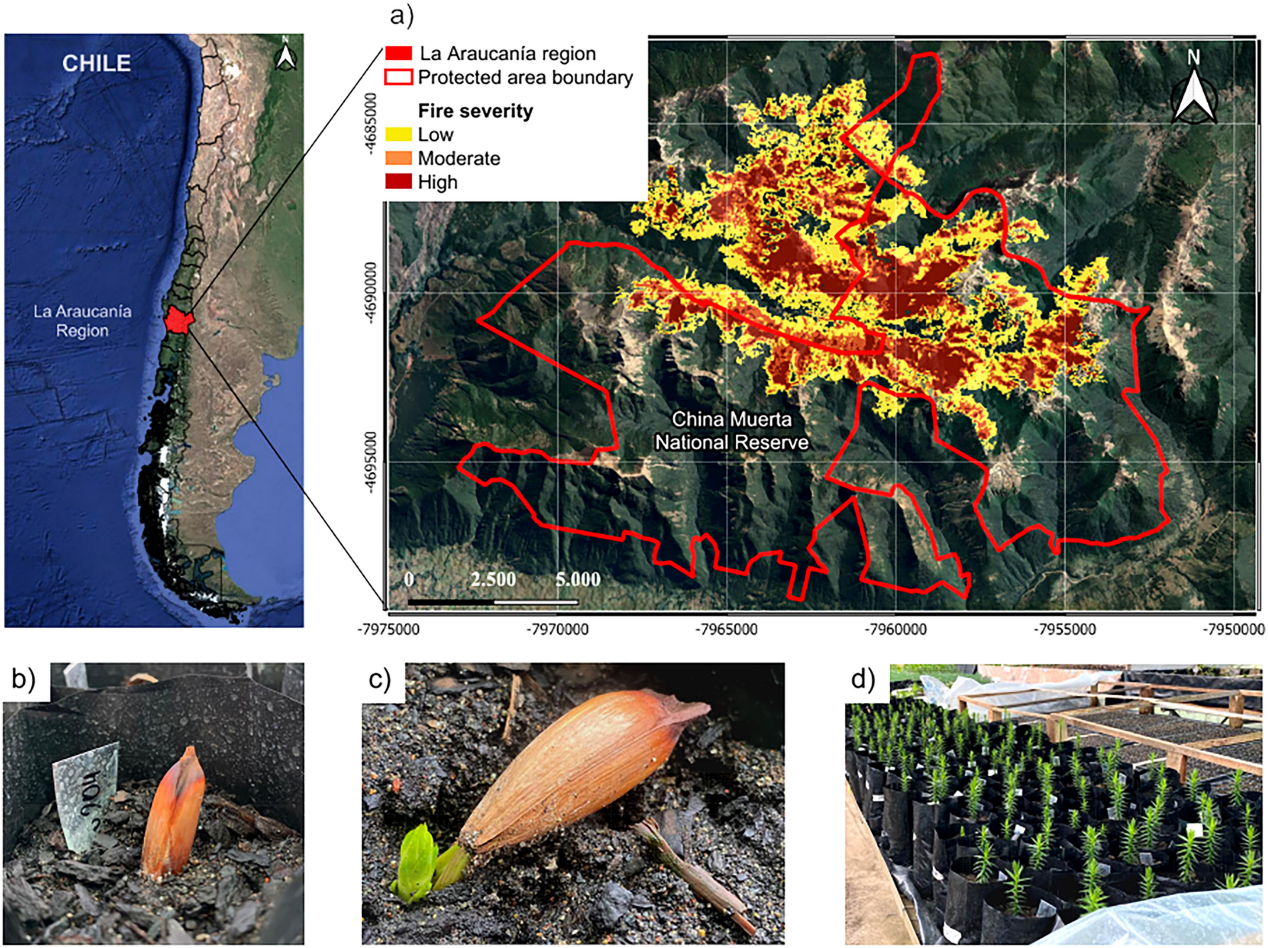
(a) Study area in the Andes of south‐central Chile with details of the burn scar of the wildfire that affected a National Reserve in 2015, where the purest stands of *A. araucana* occur. (b) Sowing of *A. araucana* seeds (locally known as *piñones* by Pehuenche communities) and (c) germination of *Araucaria* seedlings. (d) Monitoring of seedlings in the greenhouse at the end of the germination trial.

The study area is located in Andean old‐growth forests of *A. araucana* , within the National Reserve China Muerta, La Araucanía Region, south‐central Chile (38° S, 71° W, Figure 1a). Notably, along the Andes range *A. araucana* forests in this area constitute the core of the UNESCO Araucarias Biosphere Reserve. The climate is temperate, with a dry and warm summer season from December to March, and humid (i.e., with ice and snow) in winter and spring (Luebert and Pliscoff 2018), with an annual mean precipitation of 1380 mm. The maximum mean temperature in summer is 21.8°C, while the minimum mean temperature in winter is 4°C. Soils are formed from volcanic ash, with moderate depth, dark brown color, coarse texture, and good permeability (Flores et al. 2010). Vegetation is formed by temperate deciduous forests, with tree dominance of *A. araucana* (araucaria) and *N. pumilio* (lenga). The understory is mostly represented by the native species *Alstroemeria aurea* , *Chusquea culeou* , *Berberis microphylla*, and *Gaultheria poepiggi* (Urrutia‐Estrada et al. 2018).

This forest contains most of the purest stands of *A. araucana* , which were affected by a severe wildfire accidentally ignited by human activity in 2015 (March–April), affecting approximately 3750 ha (Mora and Crisóstomo 2016; Fuentes‐Ramírez et al. 2020). The fire primarily damaged forests dominated by *A. araucana* , which spread rapidly facilitated by a prolonged drought that affected south‐central Chile since 2010 (McWethy et al. 2018). The fire was classified as of mixed severity (Fuentes‐Ramírez et al. 2020), producing a mosaic of low severity areas (i.e., surface fire, greater damage to the understory), moderate‐severity areas (i.e., partially burned trees with a mix of living and dead canopy foliage), and high severity areas (i.e., canopy and understory complete charred).

### 2.2 Seed Collection and Germination Trials

Eight years after this fire, in April of 2023, seeds of *A. araucana* were collected within a 15‐m radius of previously tallied female trees assessed in 2016 (Fuentes‐Ramírez et al. 2019). These trees were not fire killed, and were selected based on the presence of visible female cones and their location in areas affected by fire, and were initially assessed in April 2016, and then in April 2023 (1 year and 8 years after fire), respectively. For this second assessment, a total of 675 seeds were randomly collected from 15 trees (i.e., 45 seeds per tree). Then, kept in plastic bags and stored in a refrigerated container at 4°C. Then, 75 seeds were randomly subjected to a cutting test, and the remaining (*n* = 600) to a flotation test to assess viability. Viable seeds (*n* = 580) were sown in 500 cc bags containing composted pine bark and sand in 3:1 ratio (Figure 1b). Fungicide treatment (Captan, 2 g/L of water) was applied at the beginning of the experiment (Piriz‐Carrillo et al. 2004; Duplancic et al. 2015), and the bags were watered once a week. These bags were tallied and placed in a greenhouse environment to monitor the germination on a weekly basis (assessed three times per week; Figure 1c) for 5 months (i.e., July–December, 2023; Figure 1d). These results were compared with data obtained in a previous experiment carried out immediately after fire by Fuentes‐Ramírez et al. (2019) using the same abovementioned protocol, where seed were collected from the same focal trees and at same time of year.

### 2.3 Statistical Analysis

The statistical analyses included estimating germination curves using the Kaplan–Meier method implemented in the R package “*survival*” (R Core Team 2025). The Kaplan–Meier curves included the 95% confidence envelopes. Also, we estimated other parameters such as germination energy (cumulative germination rate), time to peak germination (days required to reach the maximum germination rate), germination rate (ratio between the number of germinated seeds and germination time), and germination capacity (percentage of total germinated seeds plus those that have not germinated). For all this, we used the “*germinationmetrics*” package (Aravind et al. 2023), implemented in the statistical software R. Additionally, the cumulative germination curve was fitted to a specific parameter function curve (i.e., FPHF curve), which estimates the mean germination time (MGT) and the germination rate of the curve (RoG curve). Finally, we fit a parametric germination model using the “*rms*” R package to statistically compare the current data with the previous results obtained from Fuentes‐Ramírez et al. (2019) 1‐year after fire, using Likelihood Ratio (LR) test with α = 0.05.

## 3 Results

**FIGURE 2:**
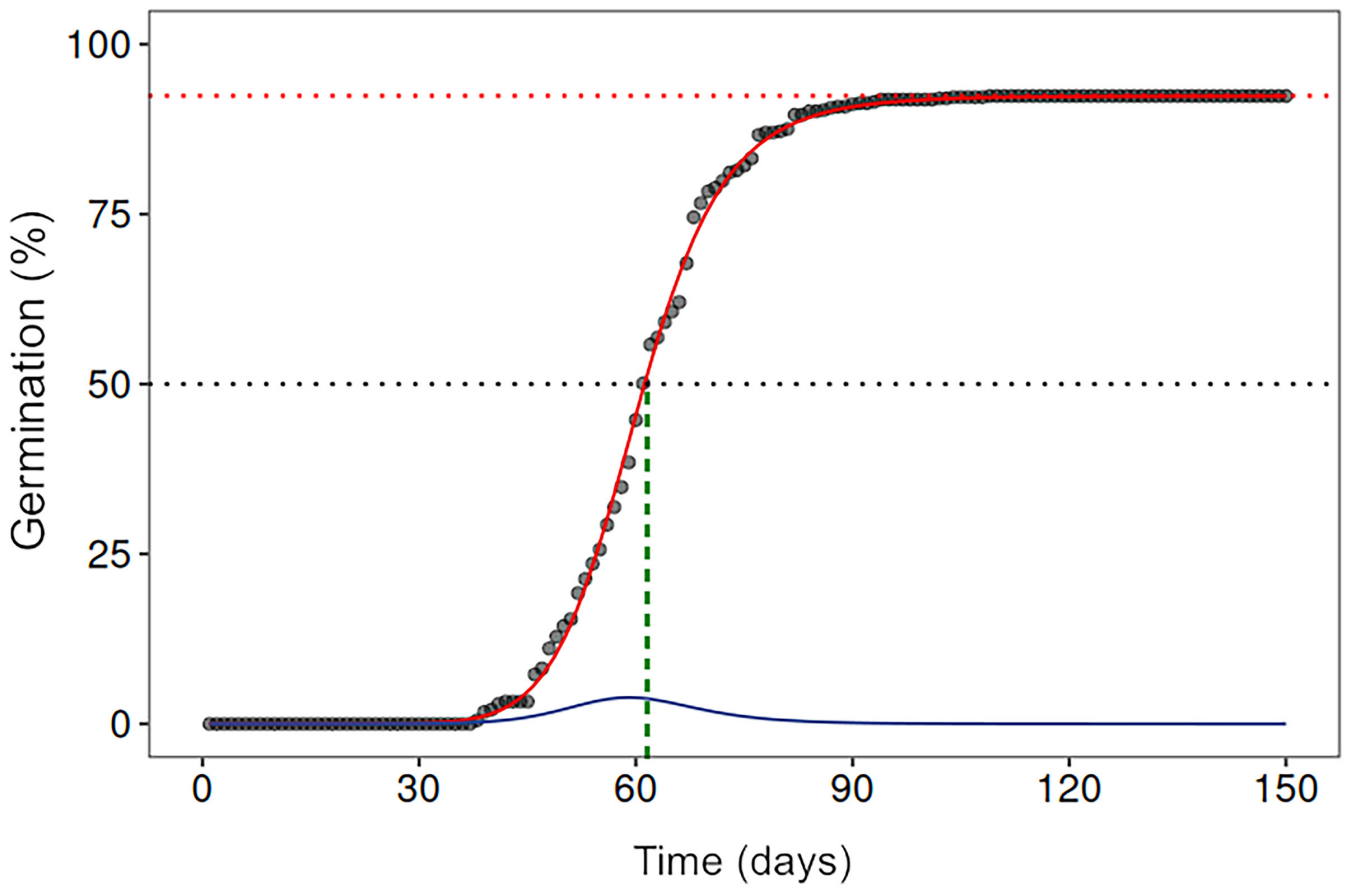
Cumulative germination curve of *A. araucana* seeds after a fire occurred 8 years ago using the hill function (FPHF) under greenhouse controlled conditions (continuous red line). The dotted black horizontal line represents the 50% germination rate and dotted red horizontal line represents the germination capacity (92.4%). The green dashed vertical line represents the mean germination time (MGT = 61.7 days). The continuous blue line represents the rate of germination (RoG) curve (i.e., germinated seeds/day).

Seeds of *A. araucana* collected 8 years after the 2015 wildfire exhibited high viability, with 90% of the seeds classified as viable based on the embryo examination test. Germination performance was also strong: of the 580 seeds sown, 533 germinated, yielding an overall germination capacity of 92%. Germination occurred over a 109‐day period, beginning on day 38 after sowing and reaching a clear peak around day 68. The mean germination speed was 3.6 seeds per day, with the fastest daily germination rate reaching 9 seeds. Modeling of germination dynamics using the Hill function (FPHF) showed high internal consistency in the temporal pattern of emergence. The time required for 50% of all seeds to germinate (T_50_) was 61.2 days, and the T_50_ calculated only for viable seeds was 60.2 days (Figure 2). The time of maximum germination rate (TMGR) was estimated at 59 days, and the mean germination time (MGT) was 61.7 days. All germination activity occurred within a 71‐day window (Figure 2). This window corresponds closely to the natural period of germination observed in the southern Andes, which typically spans from mid‐spring to early summer.

**FIGURE 3:**
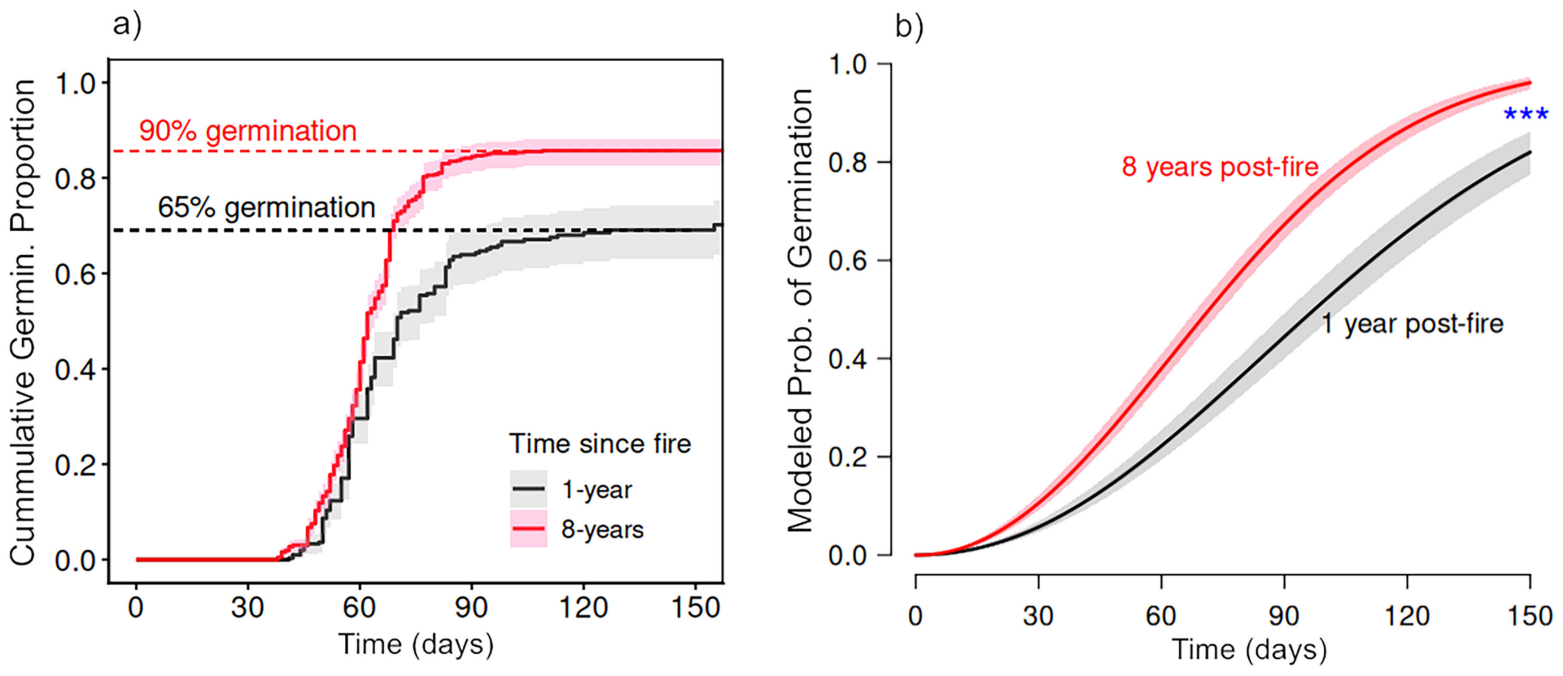
(a) Germination curves constructed with the Kaplan–Meier estimator differentiating seeds collected 1‐year and 8‐years post‐fire. (b) Modeled germination probability for seeds collected 1‐year and 8‐years post‐fire in burned *A. araucana* forests. Shaded envelopes around curves correspond to 95% confidence intervals. Note that *** in panel (b) indicates statistically significant differences between the germination curves with α = 0.05 (LR test, *χ* ^2^ = 58.99; df = 1; *p* < 0.0001).

A comparison with seeds collected 1 year after the same wildfire showed substantial differences in performance. At 1 year post‐fire, viability had reached only 70%, and germination capacity was approximately 65%, nearly 25% lower than the values observed 8 years post‐fire (Figure 3a). Statistical comparison confirmed that the germination achieved 8 years after fire was significantly higher (LR test, *χ* ^2^ = 58.99, df = 1, *p* < 0.001); (Figure 3b). The timing of germination also differed markedly: at 1 year post‐fire, germination curves showed slower emergence, lower daily germination rates, and a more extended and less defined germination period.

## 4 Discussion

Collectively, these results demonstrate substantial improvements in both germination capacity and germination speed with increasing the time since fire. This pattern suggests that, in the absence of additional disturbances, burned *A. araucana* forests retain a strong capacity for natural regeneration, reinforcing the importance of safeguarding post‐fire landscapes during early recovery phases. Germination patterns observed 8 years post‐fire contrast sharply with those recorded 1 year after fire in similar burned areas, where germination was approximately 25% lower, and seeds collected 1 year post‐fire showed 70% viability and 65% germination capacity (Fuentes‐Ramírez et al. 2019). This recovery pattern, observed under standardized greenhouse conditions that replicate the initial 2016 protocols, suggests that surviving *A. araucana* individuals retain a high capacity for physiological recovery in the absence of additional disturbances. By utilizing consistent soil substrates and controlled environments across both periods, we isolated the increase in seed quality as a function of time‐since‐fire rather than external environmental variability. These results reinforce the importance of safeguarding post‐fire landscapes during early recovery phases to allow these internal reproductive processes to stabilize.

While greenhouse conditions bypass field‐level filters like pathogens or microclimate, these controlled trials effectively isolate the intrinsic reproductive potential of Araucaria seeds. By utilizing field‐collected soil and standardized protocols, this approach provides a critical baseline for maternal recovery that is otherwise obscured by environmental stochasticity in natural sites. For instance, Donoso et al. (2024) reported 99% seed viability and 79%–92% germination when sowing *A. araucana* seeds in Andean sites with high annual precipitation (3829 mm). Comparative germination data across Araucaria species are quite limited; there is only one study examining desiccation tolerance, which grouped *A. araucana* with *A. angustifolia* and *A. bidwillii* as the most moisture‐sensitive species (requiring 25%–40% moisture content; Tompsett 1984). These similarities reinforce the ecological relevance of our findings and suggest that, when precipitation is adequate and no additional disturbances occur, *A. araucana* retains a strong intrinsic capacity for successful post‐fire regeneration.

The higher viability observed in these previous studies is expected, as neither fire severity nor the time since fire were incorporated into their assessments. For example, Sanguinetti and Kitzberger (2009) reported germination rates of 60%–95% for *A. araucana* seeds sown in *Araucaria–Nothofagus* forests of the Argentinean Andes, where annual precipitation averages 3000 mm. Likewise, Rechene et al. (2003) documented 87% germination under mean precipitation of 1047 mm, whereas Duplancic (2011) found markedly lower germination (12%–22%) when simulating xeric conditions (< 500 mm/year). These contrasting values highlight how strongly moisture availability shapes germination, but also underscore that studies explicitly evaluating post‐fire germination and particularly assessing germination over time are largely absent for this species. Together with previous studies, our findings highlight the sensitivity of *A. araucana* germination to mesic versus xeric conditions and suggest that reduced precipitation following severe fires could further constrain regeneration. Notably, climate records indicate that 2023 was wetter than preceding years (2016–2022), with above‐average precipitation (Dirección Meteorológica de Chile, DMC 2026), conditions that may have contributed to the high seed production and germination observed. Under projected increases in fire frequency and drought conditions, these factors point to compounding challenges for *A. araucana* persistence following recurrent fire events.

It is plausible that seeds collected 1 year post fire (2016) were exposed to elevated temperatures during early development, resulting in the lower viability and germination previously reported compared with seeds collected 8 years post fire. Fuentes‐Ramírez et al. (2019) found ~70% viability in seeds from recently burned areas, with embryos showing no metabolic activity, likely due to heat shock. Heat sensitivity has been demonstrated in previous studies, where constant temperatures of 35°C significantly reduced *A. araucana* germination (Duplancic et al. 2015) and brief exposure above 100°C inhibited germination entirely (Cóbar‐Carranza et al. 2015), thresholds that are easily exceeded during wildfires. Cone development further complicates fire impacts, as female cones require 2 years to mature (Hadad et al. 2021; Donoso et al. 2024). Severe or frequent fires may also reduce germination by destroying seed banks or altering soil conditions (Mataix‐Solera et al. 2021; Kasel et al. 2024). After dispersal, biological legacies such as surviving trees and downed logs can enhance recruitment by providing shade and moisture (Seidl et al. 2014; Lindenmayer et al. 2019). Thus, the protection of burned sites can improve germination success when additional disturbances are limited, whereas herbivory and seed predation may suppress recruitment (Sanguinetti and Kitzberger 2010; Zamorano‐Elgueta et al. 2012; Crovo et al. 2021). Thus, for *A. araucana* , long‐term vulnerability stems from its 2 years seed production cycle (Fuentes‐Ramírez et al. 2019), poor seed dispersal capacity, and sensitivity to short‐interval reburns that can truncate regeneration before trees reach reproductive maturity (Arroyo‐Vargas et al. 2022).

While post‐fire germination data for other Araucaria species remain limited, our results indicate that *A. araucana* exhibits a measurable increase in reproductive performance as time since fire progresses. The transition from 70% to 90% seed viability suggests that maternal recovery significantly influences seed quality in this long‐lived conifer. Consequently, these findings suggest that minimizing external pressures (i.e., grazing or seed extraction) during the initial decade post‐fire may be a prudent conservation strategy to allow these internal recovery processes to facilitate natural regeneration. Interestingly, Sanguinetti (2014) and Sanguinetti and Kitzberger (2009), through 15 years of monitoring in the region, establish that healthy *A. araucana* stands typically produce seeds with high intrinsic viability (often > 85%–90%) during reproductive cycles. Our 2024 finding of 90% germination aligns with this established species prefire norm. Consequently, the 65% germination recorded in 2016 may represent a stress‐induced deviation from the 15‐year historical average, confirming that the 8‐year interval assessed in this study allowed maternal trees to recover from fire‐induced stress and return to their characteristic reproductive potential. Furthermore, maintaining biological legacies, such as surviving trees and fallen logs, can further enhance microsites that support germination and seedling establishment (Díaz‐Mons et al. 2025). As fire frequency and severity rise across the southern Andes, incorporating post‐fire protection measures into management plans, will be crucial for safeguarding these iconic old‐growth Araucaria forests.

While our ex‐situ results demonstrate a 25% increase in intrinsic germination success, the implications for in situ regeneration are likely even more profound. In a field environment, seeds face multiple filters including seed predation, drought, and competition with opportunistic shrubs (Sanguinetti and Kitzberger 2009; Davis et al. 2018). A baseline viability of 90% provides a significantly higher buffer against these environmental stressors compared to the 65% observed immediately post‐fire. This difference suggests that the initial post‐fire window (i.e., T + 1 year) is a period of high vulnerability where lower seed quality, combined with harsh microsite conditions, could trigger recruitment failure (Fuentes‐Ramírez et al. 2019; Farid et al. 2024). Conversely, the recovery of seed quality by T + 8 years indicates that the regeneration window for these old‐growth forests may be delayed, rather than lost. Without this recovery in seed quality, there is a heightened risk of a state shift from forest to xeric woodland, particularly if subsequent disturbances or high grazing pressure occur during the vulnerable early years (Crovo et al. 2021).

## 5 Concluding Remarks

Our results demonstrate a significant increase in *A. araucana* reproductive performance over time, with seed viability rising from 70% to 90% and final germination success improving from 65% to > 90% between 1 and 8 years post‐fire. While we acknowledge that these ex‐situ findings do not account for in situ environmental filters such as granivory, soil moisture fluctuations, or microclimatic variability, this controlled evaluation is a crucial and seldom‐performed step in quantifying the recovery of the species' reproductive potential. By isolating seed performance from field‐level noise, this study sheds essential light on the recovery trajectories of maternal tree vigor and seed quality of Araucaria that are fundamental to forest resilience. These empirical insights provide a vital baseline for understanding post‐fire dynamics and underscore the importance of protecting recovering stands from anthropogenic pressures to allow these internal recovery processes to translate into successful natural regeneration.

## Author Contributions

**Andrés Fuentes‐Ramírez:** funding acquisition (lead), investigation (lead), project administration (lead), supervision (lead), writing – review and editing (equal). **Bernardita Díaz‐Mons:** data curation (lead), formal analysis (lead), writing – original draft (lead). **Rodrigo Vargas‐Gaete:** writing – review and editing (equal). **Héctor Herrera:** writing – review and editing (equal). **Leonardo Almonacid:** writing – review and editing (equal).

## Funding

This work was supported by Red Firewall initiative, ANID FOVI 220101 and ANID AMSUD 240053. ANID FONDECYT Regular, 1241295. ANID FONDEF ID25I10565. Desafíos Recuperación Post‐Incendios, ANID PINC230004. Centro ANID Basal CENAMAD, FB210015.

## Ethics Statement

Permits were granted by the Chilean Forest Service (CONAF) to carry out this research within national protected areas in the La Araucanía region (Permit 04/2024 IX).

## Conflicts of Interest

The authors declare no conflicts of interest.

## Supporting information


**Data S1:** ece374015‐sup‐0001‐DataS1.csv.

## Data Availability

All the required data are uploaded as Supporting Information.
